# Risk factors and the value of microbiological examinations of COVID-19 associated pulmonary aspergillosis in critically ill patients in intensive care unit: the appropriate microbiological examinations are crucial for the timely diagnosis of CAPA

**DOI:** 10.3389/fcimb.2023.1287496

**Published:** 2023-11-21

**Authors:** Xiaoyi Zhou, Xiaojing Wu, Ziying Chen, Xiaoyang Cui, Ying Cai, Youfang Liu, Bingbing Weng, Qingyuan Zhan, Linna Huang

**Affiliations:** ^1^ Peking University China-Japan Friendship School of Clinical Medicine, Beijing, China; ^2^ National Center for Respiratory Medicine, State Key Laboratory of Respiratory Health and Multimorbidity, National Clinical Research Center for Respiratory Diseases, Institute of Respiratory Medicine, Chinese Academy of Medical Sciences, Department of Pulmonary and Critical Care Medicine, Center of Respiratory Medicine, China-Japan Friendship Hospital, Beijing, China; ^3^ Bejing University of Chinese Medicine, Beijing, China

**Keywords:** Coronavirus Disease 2019, invasive pulmonary aspergillosis, influenza, intensive care unit, risk factors, microbiological examination

## Abstract

**Introduction:**

During the Omicron pandemic in China, a significant proportion of patients with Coronavirus Disease 2019 (COVID-19) associated pulmonary aspergillosis (CAPA) necessitated admission to intensive care unit (ICU) and experienced a high mortality. To explore the clinical risk factors and the application/indication of microbiological examinations of CAPA in ICU for timely diagnosis are very important.

**Methods:**

This prospective study included patients with COVID-19 admitted to ICU between December 1, 2022, and February 28, 2023. The clinical data of influenza-associated pulmonary aspergillosis (IAPA) patients from the past five consecutive influenza seasons (November 1, 2017, to March 31, 2022) were collected for comparison. The types of specimens and methods used for microbiological examinations were also recorded to explore the efficacy in early diagnosis.

**Results:**

Among 123 COVID-19 patients, 36 (29.3%) were diagnosed with probable CAPA. CAPA patients were more immunosuppressed, in more serious condition, required more advanced respiratory support and had more other organ comorbidities. Solid organ transplantation, APACHEII score ≥20 points, 5 points ≤SOFA score <10 points were independent risk factors for CAPA. Qualified lower respiratory tract specimens were obtained from all patients, and 84/123 (68.3%) patients underwent bronchoscopy to obtain bronchoalveolar lavage fluid (BALF) specimens. All patients’ lower respiratory tract specimens underwent fungal smear and culture; 79/123 (64.2%) and 69/123 (56.1%) patients underwent BALF galactomannan (GM) and serum GM detection, respectively; metagenomic next-generation sequencing (mNGS) of the BALF was performed in 62/123 (50.4%) patients. BALF GM had the highest diagnostic sensitivity (84.9%), the area under the curve of the mNGS were the highest (0.812).

**Conclusion:**

The incidence of CAPA was extremely high in patients admitted to the ICU. CAPA diagnosis mainly depends on microbiological evidence owing to non-specific clinical manifestations, routine laboratory examinations, and CT findings. The bronchoscopy should be performed and the BALF should be obtained as soon as possible. BALF GM are the most suitable microbiological examinations for the diagnosis of CAPA. Due to the timely and accuracy result of mNGS, it could assist in early diagnosis and might be an option in critically ill CAPA patients.

## 1 Introduction

Studies have shown that a large proportion of patients with Coronavirus Disease 2019 (COVID-19) required admission to intensive care units (ICU) (14.2-100%)(Argenziano et al., 2020; Auld et al., 2020; Cao et al., 2020; Docherty et al., 2020; Du et al., 2020). With the increase of critically ill patients, COVID-19 associated pulmonary aspergillosis (CAPA) has been paid more attention gradually. The incidence of CAPA in the ICU ranged from <1% to approximately 45% (Boyd and Martin-Loeches, 2021; Ergün et al., 2021; Autier et al., 2022; Bellanger et al., 2022; Bentvelsen et al., 2022; Erami et al., 2023). CAPA can exacerbate the course of COVID-19 and increase mortality, thereby affecting the patients’ clinical outcomes.

With extremely high mortality rate of 40-90% (Janssen et al., 2021; Segrelles-Calvo et al., 2021; van Grootveld et al., 2021; Bentvelsen et al., 2022), the timely diagnosis and treatment of severe CAPA is crucial. Therefore, it is critical to identify the clinical characteristics and risk factors of CAPA. Some studies have identified independent risk factors for CAPA, such as age, steroids, chronic pulmonary diseases, diabetes, lower lymphocyte count, and chronic renal failure (Auld et al., 2020; Machado et al., 2021; Segrelles-Calvo et al., 2021; Prattes et al., 2022). Furthermore, influenza-associated pulmonary aspergillosis (IAPA) also increases mortality and causes poor prognosis in patients admitted to ICU (Chiu and Miller, 2019; Li et al., 2020; Verweij et al., 2020; Koehler et al., 2021; Gaston et al., 2022). Therefore, exploring the similarities and differences between them might help us further explore the clinical characteristics of CAPA(Schauwvlieghe et al., 2018; Chen et al., 2020a; Coste et al., 2021).

Several studies have reported the incidence of CAPA in COVID-19 patients during the initial wave of the pandemic in China, ranging from 1% to 42.1% (Chen et al., 2020c; Song et al., 2020; Wang et al., 2020; Xu et al., 2021; Jiang et al., 2022). However, there are currently limited studies in China that investigate the clinical characteristics and risk factors of CAPA, particularly those including a control group. Furthermore, none of these studies have examined the value of microbiological examinations. Wang et al. conducted a retrospective case series involving 104 COVID-19 patients, of whom eight were diagnosed with CAPA. They identified older age, initial antibiotic combination with β-lactamase inhibitors, mechanical ventilation, and chronic obstructive pulmonary disease (COPD) as independent risk factors for CAPA(Wang et al., 2020). Another multicenter retrospective study involving 335 critically ill COVID-19 patients found that 78 (23.3%) developed CAPA. Thrombocytopenia, vasopressor use, and methylprednisolone use at a daily dose ≥ 40 mg before CAPA diagnosis were independently associated with CAPA(Xu et al., 2021). During December 2022 to February 2023, China experienced a second wave of the COVID-19 pandemic, mainly due to the omicron variant (B.1.1.529), resulting in many severe and critically ill cases, which included numerous CAPA patients. By comparing with non-CAPA patients during this pandemic and IAPA patients over the past five influenza seasons, the clinical characteristics and risk factors of critically ill CAPA patients were explored. Currently, the diagnosis of CAPA includes host factors, clinical factors, and mycological evidence, with mycological evidence playing a crucial role(Koehler et al., 2021). Among the microbiological diagnostic methods for CAPA, the recommended methods in the guidelines include fungal smear, culture, polymerase chain reaction (PCR), GM index and lateral flow assay (LFA) index, etc. PCR and LFA index were not involved in our study due to the detection methods are not widely available in China. Metagenomic next-generation sequencing (mNGS) is an emerging clinical microbiological detection method that analyzes the nucleic acid sequence of microbial pathogens in patient respiratory/blood samples and is gradually being widely used in clinical practice (Gaston et al., 2022). However, as there is no uniform standard for results interpretation, it has not been included in CAPA guidelines currently. The value of these microbiological examinations was analyzed to facilitate early diagnosis.

## 2 Materials and methods

### 2.1 Study design and patients

We included all confirmed COVID-19 patients with respiratory failure admitted to the respiratory ICU (RICU) (with 49 beds) of the China-Japan Friendship Hospital (a tertiary care center with approximately 1,700 beds) in Beijing, China, between December 1, 2022, and February 28, 2023. Clinical data of patients with influenza from the past five consecutive influenza seasons (November 1, 2017, to March 31, 2022) were collected retrospectively to determine the differences between IAPA and CAPA.

The inclusion criteria were as follows: (1) confirmed severe and critically ill COVID-19 cases, (2) over 18-years of age, and (3) eligible lower respiratory tract specimens (sputum/BALF) were obtained within 48 hours of admission to ICU and sent for mycologic tests (smear/culture/serum GM/BALF GM/histopathology). The exclusion criteria were as follows: (1) pregnancy and (2) incomplete clinical data.

The clinical characteristics and risk factors for CAPA were explored by comparing CAPA with non-CAPA patients and IAPA patients. Subsequently, the value of microbiological examinations in the diagnosis of CAPA was explored.

Demographics, clinical data, and microbiological examination results were obtained using an electronic medical record management system. The cut-off time for data collection was when all patients had clinical outcomes (April 3, 2023). Owing to the retrospective nature of the study, the need for informed consent from the patients or their legal guardians was waived.

### 2.2 Diagnostic criteria

#### 2.2.1 Definition of COVID-19, severe COVID-19 and critically ill COVID-19 cases

COVID-19 was confirmed via viral genome positivity in PCR or antigen testing. The severity of disease was defined by World Health Organization guideline for COVID-19 (WHO, 2022).

Critical COVID-19: defined by the criteria for acute respiratory distress syndrome (ARDS), sepsis, septic shock, or other conditions that would normally require the provision of life-sustaining therapies such as mechanical ventilation (invasive or non-invasive) or vasopressor therapy.

Severe COVID-19: defined by any of: oxygen saturation < 90% on room air; signs of pneumonia; signs of severe respiratory distress (in adults, accessory muscle use, inability to complete full sentences, respiratory rate > 30 breaths per minute);.

#### 2.2.2 Definition of CAPA in ICU

The diagnosis of CAPA follows the 2020 ECMM/ISHAM consensus criteria. Patients with COVID-19 needing intensive care and a temporal relationship (entry criterion) were included(Koehler et al., 2021). Probable CAPA was defined as pulmonary infiltrate, preferably documented by chest-computed tomography (CT), or cavitating infiltrate (not attributed to another cause) and at least one of the following: microscopic detection of fungal elements in bronchoalveolar lavage (BALF), indicating a mold; positive bronchoalveolar lavage culture; serum galactomannan (GM) index >0.5; bronchoalveolar lavage GM index ≥1.0. Possible CAPA was defined as pulmonary infiltrate, preferably documented by chest CT or cavitating infiltrate (not attributed to another cause) and at least one of the following: microscopic detection of fungal elements in non-bronchoscopic lavage (NBL) indicating a mold, positive NBL culture, single non-bronchoscopic lavage GM index >4.5, or NBL GM index >1.2 twice or more(Koehler et al., 2021). The final diagnosis of CAPA was made in conjunction with the consensus and the agreement of the two experienced ICU physicians.

We did not include polymerase chain reaction (PCR) and lateral flow assays (LFA) testing as diagnostic criteria for this study because we did not develop these two detection methods in our microbiology laboratory. Although metagenomic next-generation sequencing (mNGS) was not included in the diagnostic criteria, the results could generally be reported the next day, which might be helpful for early diagnosis. Therefore, for critically ill patients in ICU, we routinely conducted mNGS detection of respiratory specimens for patients with the families’ consent (Koehler et al., 2021).

#### 2.2.3 Definition of IAPA in ICU patients

The diagnosis of IAPA follows the expert case definitions of IAPA. Probable IAPA was defined as one of the following definitions: (1) confirmed influenza with pulmonary infiltration and at least one of the following: serum GM index >0.5 or BAL GM index ≥1.0 or positive BAL culture, (2) influenza was confirmed by cavitating infiltrate (not attributed to another cause) and at least one of the following: positive sputum culture or tracheal aspirate culture(Verweij et al., 2020).

### 2.3 Microbiology samples collection and processing

Serum and respiratory specimens were collected from patients for microbiological testing. BALF was acquired from patients who underwent bronchoscopy, while sputum was acquired from other patients through natural expectoration. The sputum was considered eligible when white blood cells were more than 25/low power field and epithelial cells were less than 10/low power field in sputum smears. Serum samples were sent for GM detection. Sputum specimens were sent for pathogenic smears, fungal cultures, and bacterial cultures and the BALF respiratory specimens were sent for pathogenic smears, fungal cultures, bacterial cultures, viral nucleic acid detection, and GM detection. A portion of some BALF specimens were sent for metagenomic next-generation sequencing (mNGS). Due to the high risk of bleeding for the poor coagulation function and the high risk of barotrauma for the advanced respiratory support conditions in critically ill COVID-19 patients, most patients just underwent bronchoalveolar lavage instead of lung biopsy. Meanwhile, no autopsy was conducted on the deceased patients.

Viral nucleic acid was isolated from nasal swabs in the viral transport medium with a Gene nucleic acid extraction kit (TIANLONG, Xian, China). The rRT-PCR assay was performed on the Light-Cycler480II (Switzerland) or Applied Biosystems PCR system (America).

The respiratory tract specimens were stained with fluorescent staining (Litexi, Nuoge biotechnology, China) and placed under a microscope to observe the morphology of fungal hyphae. A portion of each specimen was subcultured on Sabouraud Dextrose Agar (SDA) (Guangzhou Detgerm Microbiogical Science Ltd, China) and incubated at 28°C for 5 days. Fungal species were identified by the analysis of the mass spectrometer (MALDI-ToF, Bruker Daltonics, Germany). We consider a score of 2 or higher to be species-level reliable (Abdolrasouli and Rhodes, 2022).

Both the BALF and serum specimens were routinely sent to a microbiological laboratory for GM detection, which was performed using a double-sandwich ELISA according to the manufacturer’s instructions for the Platelia Aspergillus kit (Platelia Aspergillus; Bio-Rad, Marnes-La-Coquette, France). The optical density (OD) value for each well on the microplate reader was read, and the GM detection value in the serum or BALF samples was derived using the following formula: specimen OD value divided by the standard OD value. Serum GM index >0.5, BALF GM index ≥1.0 was defined as positive result.

Portions of the BALF specimens were sent to Vision Medical Co., Ltd. (China) (Chen et al., 2020b) or Bgi Co., Ltd. (China) (Bao et al., 2022) for mNGS analysis, nucleic acid extraction, library construction, high-throughput sequencing, bioinformatics analysis, and pathogen data interpretation.

### 2.4 Statistical analyses

Continuous variables were analyzed using the t-test and are presented as medians (inter quartile range) or mean ± standard deviation (SD). Categorical variables were analyzed using the chi-square test and are reported as numbers (%). Logistic regression analysis was conducted to identify independent risk factors for CAPA. Receiver operating characteristic (ROC) curves were plotted for the different measurement methods. These statistical analyses were performed using SPSS software version 25. The diagnostic time of various pathogen diagnostic methods was analyzed using paired t-test analysis with Prism GraphPad software, and the results were presented as a box plot chart. Statistical significance was defined as P<0.05.

## 3 Results

### 3.1 Overview of patients with severe COVID-19 and CAPA in the RICU during the Omicron pandemic

**Figure 1:**
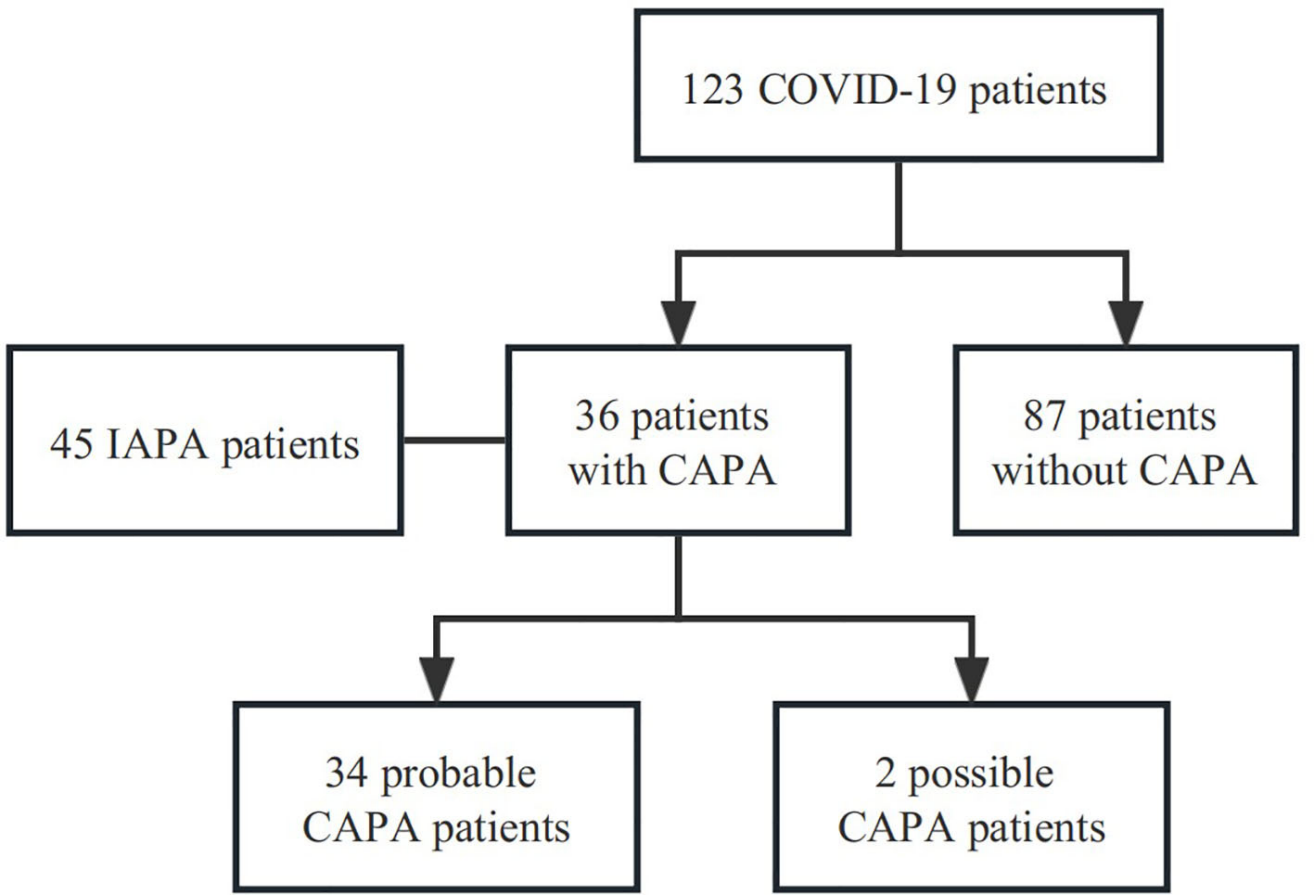
Study flowchart. A total of 123 patients with confirmed COVID-19 were admitted to the RICU between December 1, 2022, and February 28, 2023. Among them, 36 patients were diagnosed with CAPA (34 with probable CAPA, 2 with possible CAPA), while the remaining 87 patients were classified into the non-CAPA group. CAPA, COVID-19-associated pulmonary aspergillosis; IAPA, Influenza-Associated Pulmonary Aspergillosis.

**Figure 2:**
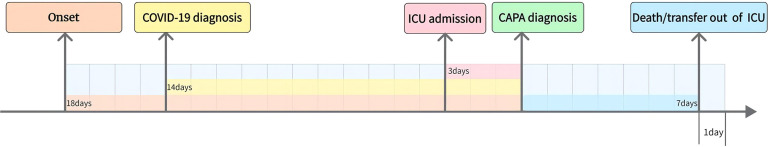
Time between onset, COVID-19 diagnosis, ICU admission, Death/transfer out of ICU and CAPA diagnosis. The time from onset, COVID-19 diagnosis and ICU admission to CAPA diagnosis was 18 (14-31) days, 14(7-25) days, and 3 (2-7) days, respectively.

A total of 123 patients with confirmed COVID-19 were admitted to the RICU between December 1, 2022, and February 28, 2023. Among them, 36 patients were diagnosed with CAPA (34 with probable CAPA, 2 with possible CAPA), while the remaining 87 patients were classified into the non-CAPA group (**Figure 1**). 2/36 (5.6%) patients were diagnosed with CAPA before ICU admission, and the median time between ICU admission and CAPA diagnosis was 3(2–7) days. Details of patients with CAPA were listed in **Table 1**. The median age of the patients was 65 (58–72) years old, of whom 86.1% (31/36) were male. 14/36 (38.9%) patients with CAPA were immunosuppressed. Overall, 29/36 (80.6%) patients received invasive mechanical ventilation (IMV), 7/36 (19.4%) received extracorporeal membrane oxygenation (ECMO) support. The median length of ICU stay was 11 days (6–26 days) and the mortality was 66.7% (24/36). The time from onset, COVID-19 diagnosis and ICU admission to CAPA diagnosis was 18 (14-31) days, 14 (7-25) days, and 3 (2-7) days, respectively. The timeline between onset, COVID-19 diagnosis, ICU admission, death/transfer out of ICU and CAPA diagnosis was shown in **Figure 2**.

**Table 1:** Diagnosis and outcomes of CAPA patients.

No.	age	Sex^1^	Immunosuppressive diseases^2^	Types of Aspergillus in sputum/BALF culture/BALF mNGS^3^	Mycological evidence^4^	Delay between ICU admission and CAPA diagnosis (days)	Highest oxygen inhalation method^5^	outcomes
1	54	M	Solid organ transplantation, Steroids and immunosuppressants in the past month	Aspergillus phoenicis	Positive BALF GM	9	IMV	Died
2	65	M	-	Aspergillus fumigatus	Positive sputum culture	7	IMV	Died
3	59	M	Solid organ transplantation, Steroids and immunosuppressants in the past month	Aspergillus flavus	Positive serum GM, BALF GM	8	IMV	Died
4	32	M	Steroidsin the past month	-	Positive BALF GM	3	IMV	Died
5	72	F	-	Aspergillus flavus, Aspergillus oryzae	Positive BALF GM, culture	5	ECMO	Died
6	62	M	Solid organ transplantation, Steroids and immunosuppressants in the past month	Aspergillus fumigatus, Aspergillus flavus	Positive BALF culture	10	IMV	Died
7	53	M	Solid organ transplantation, Steroids and immunosuppressants in the past month	Aspergillus fumigatus, Aspergillus niger, Aspergillus flavus	Positive BALF GM and culture	2	HFNC	Survived
8	56	M	-	Aspergillus fumigatus	Positive BALF GM	3	IMV	Died
9	65	M	-	Aspergillus tubingensis	Positive BALF GM	3	IMV	Survived
10	62	M	Solid organ transplantation, Steroids and immunosuppressants in the past month	Aspergillus fumigatus	Positive BALF culture	7	IMV	Died
11	69	F	Solid organ transplantation, Solid Malignant Tumor,Steroids and immunosuppressants in the past month	-	Positive serum GM, BALF GM	-1	ECMO	Died
12	48	M	Solid organ transplantation, Steroids and immunosuppressants in the past month	Aspergillus fumigatus, Aspergillus terrestris	Positive BALF GM, culture	2	ECMO	Died
13	59	M	Solid organ transplantation, Steroids and immunosuppressants in the past month	-	Positive serum GM, BALF GM	3	IMV	Died
14	45	M	Solid organ transplantation, Steroids and immunosuppressants in the past month	Aspergillus flavus	Positive serum GM	-16	IMV	Died
15	67	M	-	Aspergillus fumigatus, Aspergillus flavus	Positive serum GM, BALF GM	2	IMV	Survived
16	70	M	-	Aspergillus fumigatus, Aspergillus niger	Positive BALF GM	1	IMV	Died
17	61	M	-	Aspergillus fumigatus	Positive BALF GM	2	ECMO	Died
18	58	M	-	-	Positive serum GM, BALF GM	26	ECMO	Survived
19	73	M	-	Aspergillus fumigatus, Aspergillus flavus, Aspergillus terrestris	Positive BALF GM and culture	1	ECMO	Survived
20	71	F	-	Aspergillus fumigatus	Positive serum GM, BALF GM and culture	5	IMV	Survived
21	63	M	-	Aspergillus fumigatus	Positive BALF culture	8	ECMO	Died
22	79	M	-	Aspergillus fumigatus	Positive BALF GM and culture	2	IMV	Died
23	76	M	-	Aspergillus fumigatus	Positive serum GM, BALF culture	5	IMV	Died
24	88	M	Solid Malignant Tumor	Aspergillus niger	Positive BALF GM and culture	14	HFNC	Died
25	77	M	-	Aspergillus flavus, Aspergillus terrestris	Positive BALF GM and culture	3	IMV	Died
26	62	M	.Solid organ transplantation, Immunosuppressants in the past month	Aspergillus fumigatus	Positive BALF GM and culture	4	Nasal catheter	Survived
27	84	M	-	Aspergillus fumigatus	Positive BALF GM and culture	4	HFNC	Survived
28	69	M	-	-	Positive serum GM, BALF GM	7	IMV	Died
29	69	M	-	Aspergillus fumigatus	Positive BALF GM and culture	2	IMV	Died
30	73	M	-	-	Positive serum GM	24	NIMV	Survived
31	57	M	Solid organ transplantation, Steroids and immunosuppressants in the past month	Aspergillus fumigatus	Positive BALF GM and culture	10	IMV	Survived
32	78	F	Steroids and immunosuppressants in the past month	-	Positive BALF GM	7	HFNC	Survived
33	70	M	-	-	Positive BALF GM	3	IMV	Died
34	64	M	-	-	Positive BALF GM	3	IMV	Died
35	76	F	Hematological malignancies, Steroids and immunosuppressants in the past month	Aspergillus fumigatus	Positive sputum culture	1	NIMV	Survived
36	48	M	Solid organ transplantation, Steroids and immunosuppressants in the past month	-	Positive serum GM, BALF GM	0	IMV	Died

1. M, Male; F, Female.

2. ‘-’ means not had other host factors.

3. ‘-’ means not had Positive results.

4. Positive serum GM: serum GM index>0.5; Positive BALF GM: BALF GM index≥1.0.

5. IMV, Invasive mechanical ventilation; NIMV, Non-invasive mechanical ventilation; ECMO, Extra Corporeal Membrane Oxygenation; HFNC, High Flow Nasal Cannula.

Regarding antifungal therapy, 21/36 (58.3%) patients were treated with voriconazole monotherapy as initial therapy. In total, 4/36 (11.1%) patients were treated with amphotericin B or caspofungin without voriconazole in the early stage because of the use of paxlovid and switched to/added voriconazole later. Only one patient (2.7%) was initially treated with caspofungin because of combination with Candida albicans infection. Overall, 3/36 (8.3%) patients were treated in combination with amphotericin B or caspofungin because of exacerbation during treatment and 3/36 (8.3%) patients were switched to caspofungin or isavuconazole as the salvage therapy because of the side effects (liver damage/renal damage) of voriconazole.

### 3.2 Explore the clinical characteristics and risk factors in critically ill patients with CAPA

#### 3.2.1 Comparisons between CAPA and non‐CAPA patients

As is shown in **Table 2**, patients with solid organ transplantation (33.3% vs. 12.6%,p=0.007) were higher in the CAPA group. More patients with CAPA use steroids before ICU admission (80.6% vs. 60.9%, p=0.019), and the total amount of steroids administered was also higher (237 mg vs. 50 mg of equivalent prednisone, p=0.007). CAPA patients were in a more severe condition than non-CAPA patients which manifested as higher Acute Physiology and Chronic Health Evaluation (APACHE) II scores (20 vs. 15 points, p=0.017) and Sequential Organ Failure Assessment (SOFA) scores (7 vs. 4 points, p=0.024).

Respiratory symptoms did not differ between the two groups. Most routine laboratory examinations show no difference between the two groups, with the exception of higher IL-6 levels (86.86 vs. 25.11 pg/mL, p= 0.017), higher IL-8 levels (18.32 vs. 12.48 pg/mL, p=0.024), lower CD4+ T lymphocytes (156 vs. 205 cells/µl, p=0.042), and lower CD8+ T (82 vs. 142 cells/µl, p=0.006) lymphocytes in CAPA group. Chest CT scans were performed in all patients within 5 days of CAPA diagnosis. Bronchial wall thickening (72.2% vs. 46.0%, p=0.008) was observed more often in CAPA patients than in non-CAPA patients, whereas no significant differences were observed in nodules, halo sign, cavitation, wedge-shaped solid lesions, and tree-in-bud sign (**Table 2**).

More patients with CAPA received IMV (80.6% vs. 49.4%; p=0.001), tracheotomy (52.8% vs. 27.6%; p=0.008), prone position (52.8% vs. 27.6%; p=0.008), or ECMO support (19.4% vs. 4.6%, p=0.023) compared with the control group. Furthermore, patients with CAPA were more likely to combine with bacterial infections (80.6% vs. 43.7%, p<0.001), viral infections (38.9% vs. 14.9%, p=0.004), other fungal infections (50.0% vs. 28.7%, p=0.024), and had higher rate of complications during ICU admission, including hospital acquired pneumonia (58.3% vs. 25.3%, p<0.001), catheter related bloodstream infection (8.3% vs. 0, p=0.024), acute renal injury (47.2% vs. 25.3%, p=0.017), circulatory failure (52.8% vs. 31.0%, p=0.023), hematological system failure (47.2% vs. 23.0%, p=0.008), and pulmonary embolism (8.3% vs. 0, p=0.024). Although the mortality rate of patients with CAPA was extremely high, there was no significant difference between the two groups (**Table 2**).

**Table 2:** Comparisons between CAPA and non-CAPA patient.

Variable	Total(N=123)	CAPA(n=36)	Non-CAPA (n=87)	P
Age (years,median,IQR)	69 (59-78)	65 (58-72)	69 (59-80)	0.126
Male, n (%)	98 (79.7%)	31 (86.1%)	67 (77.0%)	0.254
BMI (kg/m2,mean ± SD)	24.41 ± 3.67	23.38 ± 4.03	24.84 ± 3.44	0.043
Smoke, n (%)	42 (34.1%)	12 (33.3%)	30 (34.5%)	0.903
Drink,n (%)	28 (22.8%)	10 (27.8%)	18 (20.7%)	0.394
Underlying disease, n (%)				
Diabetes	49 (39.8%)	18 (50.0%)	31 (35.6%)	0.139
Hypertension	74 (60.2%)	23 (63.9%)	51 (58.6%)	0.587
Chronic lung disease	18 (14.6%)	6 (16.7%)	12 (13.8%)	0.682
Chronic heart failure	16 (13.0%)	3 (8.3%)	13 (14.9%)	0.321
Chronic renal insufficiency	27 (22.0%)	12 (33.3%)	15 (17.2%)	0.050
Immunosuppression status, n (%)				
Any immunosuppressive condition	49 (39.8%)	14 (38.9%)	35 (40.2%)	0.890
Solid organ transplantation	23 (18.7%)	12 (33.3%)	11 (12.6%)	0.007
Solid Malignant Tumor	8 (6.5%)	2 (5.6%)	6 (6.9%)	1.000
Hematological malignancies	5 (4.1%)	1 (2.8%)	4 (4.6%)	1.000
Steroid in the past month (not for COVID-19)	29 (23.6%)	9 (25.0%)	20 (23.0%)	0.811
Steroid pulse therapy in the past month	7 (5.7%)	2 (5.6%)	5 (5.7%)	1.000
Daily average dosage of steroid in the past month (not for COVID-19/influenza) (equal amount of prednisone, mg,median,IQR)	0 (0-0)	0 (0-3.75)	0 (0-0)	0.855
Immunosuppressants in the past month	42 (34.1%)	13 (36.1%)	29 (33.3%)	0.768
COVID-19 related treatment before admission to ICU, n(%)				
Antiviral drugs	45 (36.6%)	20 (55.6%)	25 (28.7%)	0.005
Paxlovid	37 (30.1%)	15 (41.7%)	22 (25.3%)	0.072
Azvudine	11 (8.9%)	6 (16.7%)	5 (5.7%)	0.053
Ostavir	6 (4.9%)	2 (5.6%)	4 (4.6%)	0.822
Antibacterial drugs	103 (83.7%)	35 (97.2%)	68 (78.2%)	0.019
Antifungal drugs	9 (7.3%)	4 (11.1%)	5 (5.7%)	0.510
Steroids	82 (66.7%)	29 (80.6%)	53 (60.9%)	0.036
Steroids use≥7days	32 (26.0%)	18 (20.7%)	14 (38.9%)	0.036
Total dosage of steroid in the past month for COVID-19/influenza (equal amount of prednisone, mg,median,IQR)	100 (0-300)	237 (62.5-487.5)	50 (0-290)	0.007
Intra-Venous ImmunoGlobulin (IVIG)	19 (15.4%)	9 (25.0%)	10 (11.5%)	0.059
Anticoagulants	41 (33.3%)	11(30.6%)	30 (34.5%)	0.674
Disease severity score on ICU admission				
APACHEII score (points,median,IQR)	16 (12-22)	20 (13-25)	15 (11-21)	0.017
APACHEII≥20scores	43 (35.0%)	18 (50.0%)	25 (28.7%)	0.024
SOFA score (points, median,IQR)	5 (3-8)	7(5-9)	4 (3-8)	0.024
SOFA score<5points	53 (43.1%)	8 (22.2%)	45 (51.7%)	0.001
5points≤SOFA score<10points	44 (35.8%)	22 (61.1%)	22 (25.3%)	
SOFA score≥10points	26 (21.1%)	6 (16.7%)	20 (23.0%)	
Symptoms, n (%)				
Fever	114 (92.7%)	36 (100%)	78 (89.7%)	0.057
Tmax (°C,median,IQR)	38.5 (38.0-39.0) n=101	38.4 (37.5-39.0) n=33	38.5 (38.0-39.0) n=68	0.429
Cough	98 (79.7%)	30 (83.3%)	68 (78.2%)	0.517
Expectoration	86 (69.9%)	25 (69.4%)	61 (70.1%)	0.941
Dyspnea	113 (91.9%)	35 (97.2%)	78 (89.7%)	0.301
hemoptysis	1 (0.8%)	0	1 (1.1%)	1.000
Sign				
Temperature (°C,median,IQR)	36.8 (36.5-37.2)	36.7 (36.5-37.0)	36.8 (36.5-37.2)	0.967
Respiration≥30 times/minute	26 (21.1%)	13 (36.1%)	13 (14.9%)	0.009
Heart rate >100 (beats per minute,median,IQR)	37 (30.1%)	10 (27.8%)	27 (31.0%)	0.720
Blood gas analysis				
pH (median,IQR)	7.42 (7.36-7.47)	7.42 (7.34-7.44)	7.43 (7.37-7.47)	0.097
PaO_2_/FiO_2_<100 (mmHg,median,IQR)	60 (48.8%)	19 (52.8%)	41 (47.1%)	0.568
Laboratory examination				
WBC (*10^9/L,median,IQR)	9.17 (5.96-12.66)	9.51 (5.65-13.68)	8.83 (5.99-12.53)	0.611
LYM (*10^9/L,median,IQR)	0.47 (0.26-0.69)	0.50 (0.23-0.64)	0.47 (0.28-0.72)	0.455
D-D (ng/mL,median,IQR)	2.48 (1.12-11.23)	4.63 (0.88-18.11)	2.30 (1.24-8.23)	0.368
CRP (mg/L,median,IQR)	85.63 (43.77-164.98)	87.59 (42.98-187.39)	85.63 (43.77-148.35)	0.740
PCT (mg/L,median,IQR)	0.23 (0.10-0.84)	0.26 (0.10-0.94)	0.22 (0.10-0.81)	0.647
ESR (mm/h,mean ± SD)	44.13 ± 27.26 N=54	51.7 ± 29.2 N=17	40.7 ± 26.0 N=37	0.168
Fet (ng/L,median,IQR)	646.30 (397.80-964.85) N=113	662.7 (389.4-1149.3) N=34	639.2 (403.3-955.7) N=79	0.858
CT value of COVID-19<30	65 (52.8%)	23 (63.9%)	42 (48.3%)	0.115
Cytokine				
IL-6 (pg/mL,median,IQR)	35.02 (10.98-131.96)	86.86 (14.56-416.40)	25.11 (10.00-86.70)	0.017
IL-8 (pg/mL,median,IQR)	13.44 (7.35-32.27)	18.32 (9.95-42.97)	12.48 (6.19-27.95)	0.024
IL-10 (pg/mL,median,IQR)	2.54 (2.44-5.58)	3.07 (2.44-7.78)	2.52 (2.44-5.10)	0.331
TNF-α (pg/mL,median,IQR)	2.44 (2.44-2.44)	2.44 (2.44-2.44)	2.44 (2.44-2.44)	0.609
Immunocyte				
CD4^+^ T lymphocytes (cells/,median,IQR)	192 (123-296)	156 (91-275)	205 (134-328)	0.042
CD8^+^ T lymphocytes (mg/L,median,IQR)	124 (74-202)	82 (54-145)	142 (80-217)	0.006
CD4^+^/CD8^+^(median,IQR)	1.67 (1.03-2.44)	1.73 (0.96-2.74)	1.65 (1.03-2.29)	0.298
Serum GM (highest)	0.25 (0.11-0.38) n=66	0.33 (0.20-1.42) n=26	0.17 (0.09-0.31) n=40	0.001
BALG GM (highest)	0.79 (0.21-3.12) n=79	4.86 (1.81-7.28) n=33	0.20 (0.26-0.59) n=46	<0.001
Positive G test	7 (5.7%)	4 (11.1%)	3 (3.4%)	0.214
CT imaging, n (%)				
signs of vascular invasion	32 (26.0%)	13 (36.1%)	19 (21.8%)	0.101
Cavity/air crescent sign	3 (2.4%)	2 (5.6%)	1 (1.1%)	0.424
Soft tissue shadow	2 (1.6%)	1 (2.8%)	1 (1.1%)	1.000
Wedge-shaped segmental	10 (8.1%)	4 (11.1%)	6 (6.9%)	0.678
Halo sign	28 (22.8%)	11 (30.6%)	17 (19.5%)	0.185
signs of airway invasion	79 (64.2%)	28 (77.8%)	51 (58.6%)	0.044
Central lobular nodules	4 (3.3%)	2 (5.6%)	2 (2.3%)	0.713
Patches distributed along the airway	33 (26.8%)	8 (22.2%)	25 (28.7%)	0.458
Bronchial wall thickening	66 (53.7%)	26 (72.2%)	40 (46.0%)	0.008
Tree in bud	5 (4.1%)	3 (8.3%)	2 (2.3%)	0.298
Ground glass opacity	109 (88.6%)	32 (88.9%)	77 (88.5%)	1.000
Grid shadow	41 (33.3%)	9 (25.0%)	32 (36.8%)	0.207
Consolidation	44 (35.8%)	17 (47.2%)	27 (31.0%)	0.088
Pleural effusion	41 (33.3%)	9 (25.0%)	32 (36.8%)	0.207
Mediastinal lymph node enlargement	14 (11.4%)	2 (5.6%)	12 (13.8%)	0.319
Pneumothorax	1 (0.8%)	1 (2.8%)	0	0.293
Mediastinal emphysema	3 (2.4%)	1 (2.8%)	2 (2.3%)	1.000
Treatments during ICU stay				
Tracheal intubation,n (%)	71 (57.7%)	29 (80.6%)	43 (49.4%)	0.001
Tracheotomy,n (%)	43 (35.0%)	19 (52.8%)	24 (27.6%)	0.008
ECMO, n (%)	11 (8.9%)	7 (19.4%)	4 (4.6%)	0.023
Prone position, n (%)	43 (35.0%)	19 (52.8%)	24 (27.6%)	0.008
Pulmonary recruitment, n (%)	7 (5.7%)	3 (8.3%)	4 (4.6%)	0.700
CRRT, n (%)	33 (26.8%)	13 (36.1%)	20 (23.0%)	0.135
Paxlovid, n (%)	68 (55.3%)	24 (66.7%)	44 (50.6%)	0.102
Steroid, n (%)	110 (89.4%)	33 (91.77%)	77 (88.5%)	0.844
Tolimumab, n (%)	21 (17.1%)	9 (25.0%)	12 (13.8%)	0.133
Baricitinib, n (%)	19 (15.4%)	6 (16.7%)	13 (14.9%)	0.810
Tofacitab,n (%)	3 (2.4%)	0	3 (3.4%)	0.555
Anticoagulation,n (%)	117 (95.1%)	35 (97.2%)	82 (94.3%)	0.670
Antibacterial,n (%)	117 (95.1%)	36 (100%)	81 (93.1%)	0.179
Anti-fungal,n (%)	61 (49.6%)	32 (88.9%)	34 (39.1%)	<0.001
Complications and outcomes,n (%)				
Bacterial infection,n (%)	67 (54.5%)	29 (80.6%)	38 (43.7%)	<0.001
Viral infection,n (%)	27 (22.0%)	14 (38.9%)	13 (14.9%)	0.004
Fungal infections other than Aspergillus,n (%)	43(35.0%)	18 (50.0%)	25 (28.7%)	0.024
Barotrauma,n (%)	13 (10.6%)	6 (16.7%)	7 (8.0%)	0.157
Hospital acquired pneumonia,n (%)	43 (35.0%)	21 (58.3%)	22 (25.3%)	<0.001
Catheter Related Bloodstream Infection,n (%)	3(2.4%)	3 (8.3%)	0	0.024
Abdominal infection,n (%)	2 (1.6%)	1 (2.8%)	1 (1.1%)	1.000
Bloodstream infection,n (%)	9 (7.3%)	5 (13.9%)	4 (4.6%)	0.156
Acute renal failure,n (%)	36 (29.3%)	17 (47.2%)	22 (25.3%)	0.017
Circulatory failure,n (%)	46 (37.4%)	19 (52.8%)	27 (31.0%)	0.023
Hepatic failure,n (%)	11 (8.9%)	4 (11.1%)	7 (8.0%)	0.846
Hematological system failure,n (%)	37 (30.1%)	17 (47.2%)	20 (23.0%)	0.008
Central system failure,n (%)	5 (4.1%)	2 (5.6%)	3 (3.4%)	0.971
Deep vein thrombosis,n (%)	28 (22.8%)	10 (27.8%)	18 (20.7%)	0.394
Pulmonary embolism,n (%)	3 (2.4%)	3 (8.3%)	0	0.024
Gastrointestinal bleeding,n (%)	29 (23.6%)	11 (30.6%)	18 (20.7%)	0.241
Length of invasive ventilation (days,median,IQR)	11 (5-21) N=72	12 (5-26) N=29	10 (5-17) N=43	0.418
ICU Mortality,n (%)	68 (55.3%)	24 (66.7%)	44 (50.6%)	0.102
Length of ICU (days,median,IQR)	10 (5-21)	11 (6-26)	10 (4-19)	0.104
Length of hospitalization (days,median,IQR)	21 (12-30)	24 (13-40)	18 (12-27)	0.055

IQR, Interquartile Range; BMI, Body Mass Index; SD , Standard Deviation; APACHE, Acute Physiology and Chronic Health Evaluation; SOFA, sequential organ failure assessment; WBC, white blood cell; LYM, lymphocyte; CRP, C-reactive protein; PCT, procalcitonin; ESR, erythrocyte sedimentation rate; ECMO, extracorporeal membrane oxygenation; CRRT, continuous renal replacement therapy; Circulatory failure: dopamine>5 or epinephrine>0.1 or norepinephrine>0.1; Hepatic failure: bilirubin>5.9mg/dL(101 μmol/L); Hematological system failure:platelet<50*10^9/L; Central system failure: Glasgow Coma Scale ≤ 9 points.

#### 3.2.2 Comparisons between CAPA and IAPA patients

In total, 45 IAPA patients were included in our study and compared to patients with CAPA. Compared to IAPA patients, the time from onset to CAPA diagnosis, from COVID-19/influenza diagnosis and CAPA/IAPA diagnosis and from ICU admission to CAPA diagnosis was longer (**Table 3**).

Compared to IAPA, less CAPA smoked, and more CAPA patients had renal insufficiency (33.3% vs. 6.7%, p=0.005) and solid organ transplantation (33.3% vs. 4.4%, p=0.002). More patients with CAPA had used immunosuppressants (37.8% vs. 11.1%, p=0.004) within one month prior to ICU admission. More patients with CAPA used steroids for COVID-19 within one month before ICU admission than IAPA (80.6% vs. 40.0%, p<0.001). There was also a significant difference between the two groups in terms of the accumulated steroid dosage in the past month (CAPA vs. IAPA: 237 vs. 0 mg of equivalent prednisone, p<0.001).

There were no significant differences in the positive rate and absolute values of serum GM, BALF GM, and G test results between the two groups. Regarding CT scans, CAPA patients showed a lower percentage of cavity/air crescent sign (5.4% vs. 37.8%, p=0.002), wedge-shaped solid change (11.1% vs. 42.2%, p=0.004), central lobular nodules (5.6% vs. 42.2%, p<0.001), patchiness distributed along the airway (22.2% vs. 88.9%, p<0.001), and tree-in-bud sign (8.3% vs. 33.3%, p=0.013) compared to IAPA. Patient with CAPA were much more likely to use steroids after ICU admission compared with patients with IAPA (91.7% vs. 11.1%, p<0.001). The clinical outcomes were also similar, with no significant differences in mortality, length of ICU stay, or length of hospitalization (**Table 3**).

**Table 3:** Comparisons between CAPA and IAPA patients.

Variable	Total (n=81)	CAPA (n=36)	IAPA (n=45)	P
Age (years,mean ± SD)	62 ± 15	65 ± 11	60 ± 17	0.121
Male, n (%)	68 (84.0%)	31 (86.1%)	37 (82.2%)	0.636
BMI (kg/m2,mean ± SD)	23.71 ± 3.99	23.38 ± 4.03	23.98 ± 3.98	0.505
Smoke,n (%)	37 (45.7%)	12 (33.3%)	25 (55.6%)	0.046
Drink,n (%)	26 (32.1%)	10 (27.8%)	16 (35.6%)	0.456
Underlying disease,n (%)				
Diabetes	34 (42.0%)	18 (50.0%)	16 (35.6%)	0.191
Chronic lung disease	21 (25.9%)	6 (16.7%)	15 (33.3%)	0.089
Chronic cardiac insufficiency	9 (11.1%)	3 (8.3%)	6 (13.3%)	0.722
Renal insufficiency	15 (18.5%)	12 (33.3%)	3 (6.7%)	0.005
Liver dysfunction	3 (3.7%)	2 (5.6%)	1 (2.2%)	0.844
Connective tissue disease	5 (6.2%)	1 (2.8%)	4 (8.9%)	0.502
Immunosuppression status,n (%)				
Solid organ transplantation	14 (17.3%)	12 (33.3%)	2 (4.4%)	0.002
Solid Malignant Tumor	4 (4.9%)	2 (5.6%)	2 (4.4%)	1.000
Hematological malignancies	2 (2.5%)	1 (2.8%)	1 (2.2%)	1.000
Immunosuppressants in the past month	18 (22.2%)	13 (36.1%)	5 (11.1%)	0.007
Steroid in the past month (not for COVID-19/influenza)	26 (32.1%)	11 (30.6%)	15 (33.3%)	0.790
Daily average dosage of steroid in the past month (not for COVID-19/influenza) (equal amount of prednisone, mg, median, IQR)	0 (0-0)	0 (0-3.75)	0 (0-0)	0.535
Steroid in the past month (for COVID-19/influenza)	47 (58.0%)	29 (80.6%)	18 (40.0%)	<0.001
Total dosage of steroid in the past month for COVID-19/influenza (equal amount of prednisone, mg,median,IQR)	50 (0-273.5)	237 (62.5-487.5)	0 (0-109)	<0.001
Time between onset and CAPA/IAPA diagnosis (days,median,IQR)	17 (12-22)	18 (14-31)	14 (9-21)	0.002
Time between COVID-19/influenza diagnosis and CAPA/IAPA diagnosis (days,median,IQR)	5 (1-14)	14 (7-25)	1 (0-7)	<0.001
Time between ICU admission and CAPA/IAPA diagnosis (days,median,IQR)	2 (1-5)	3 (2-7)	2 (1-3)	0.001
Time between CAPA/IAPA diagnosis and death/ICU leave (days,median,IQR)	11 (4-28)	7 (1-24)	13 (6-35)	0.072
Symptoms,n (%)				
Fever	74 (90.1%)	36 (100%)	37 (82.2%)	0.008
Cough	73 (90.1%)	30 (83.3%)	43 (95.6%)	0.145
Expectoration	62 (76.5%)	25 (69.4%)	37 (82.2%)	0.177
Dyspnea	76 (93.8%)	35 (97.2%)	41 (91.1%)	0.502
Chest pain	6 (7.4%)	2 (5.6%)	4 (8.9%)	0.887
hemoptysis	10 (12.3%)	0	10 (22.2%)	0.002
Sign				
Respiration (beats per minute, mean ± SD)	27 ± 6	26 ± 6	28 ± 6	0.269
Heart rate (beats per minute, median,IQR)	91 ± 21	89 ± 28	92 ± 14	0.530
Blood gas analysis				
pH (median,IQR)	7.42 (7.31-7.45)	7.42 (7.34-7.44)	7.42 (7.29-7.47)	0.936
PaCO_2_ (mmHg,median,IQR)	40.4 (33.2-54.2)	37.6 (32.9-46.8)	43.4 (33.4-58.8)	0.300
PaO_2_/FiO_2_ (mmHg,median,IQR)	132 (92-195)	97 (70-162)	151 (109-221)	0.002
Laboratory examination				
WBC (*10^9/L,median,IQR)	10.97 (5.75-15.81)	9.51 (5.65-13.68)	11.90 (6.47-18.14)	0.091
LYM (*10^9/L,median,IQR)	0.59 (0.32-0.77)	0.50 (0.23-0.64)	0.68 (0.39-0.90)	0.004
PCT (mg/L,median,IQR)	0.42 (0.20-1.70)	0.26 (0.10-0.94)	0.83 (0.24-2.43)	0.009
CD4^+^ T lymphocytes (cells/μl,median,IQR)	189 (104-329)	158 (81-294)	198 (117-371)	0.119
CD8^+^ T lymphocytes (cells/μl,median,IQR)	127 (59-227)	83 (50-160)	153 (104-252)	0.019
CD4^+^/CD8^+^ (median,IQR)	1.67 (0.87-2.68)	1.75 (0.83-2.89)	1.62 (0.94-2.60)	0.528
Serum GM>0.5 (the first time)	28 (39.4%) n=71	10 (38.5%) n=26	18 (40.0%)	0.898
Serum GM>0.5 (highest)	33 (46.5%) n=71	11 (42.3%) n=26	22 (48.9%)	0.592
BALG GM≥1 (the first time)	57 (73.1%) n=78	24 (72.7%) N=33	33 (73.3%)	0.952
BALG GM≥1 (highest)	65 (83.3%) n=78	28 (84.8%) n=33	37 (82.2%)	0.758
Positive G test	12 (14.8%)	4 (11.1%)	8 (17.8%)	0.600
CT manifestations,n (%)				
signs of vascular invasion	39 (48.8%)	13 (36.1%)	26 (59.1%)	0.041
Cavity/air crescent sign	19 (23.5%)	2 (5.4%)	17 (37.8%)	0.002
Soft tissue shadow	2 (2.5%)	1 (2.8%)	1 (2.2%)	1.000
Wedge-shaped solid change	23 (28.4%)	4 (11.1%)	19 (42.2%)	0.005
Halo sign	27 (33.3%)	11 (30.6%)	16 (35.6%)	0.635
signs of airway invasion	70 (87.5%)	28 (77.8%)	42 (95.5%)	0.041
Central lobular nodules	21 (25.9%)	2 (5.6%)	19 (42.2%)	<0.001
Patches distributed along the airway	48 (59.3%)	8 (22.2%)	40 (88.9%)	<0.001
Thickening of air duct wall	63 (77.8%)	26 (72.2%)	37 (82.2%)	0.282
Tree bud sign	18 (22.2%)	3 (8.3%)	15 (33.3%)	0.013
Ground glass opacity	65 (80.2%)	32 (88.9%)	33 (73.3%)	0.143
Grid shadow	22 (26.7%)	10 (27.8%)	12 (26.7%)	0.911
Pleural effusion	27 (33.3%)	9 (25.0%)	18 (40.0%)	0.155
Mediastinal lymph node enlargement	12 (14.8%)	2 (5.6%)	10 (22.2%)	0.075
Pneumothorax	3 (3.7%)	1 (2.8%)	2 (4.4%)	1.000
Mediastinal emphysema	5 (6.2%)	1 (2.8%)	4 (8.9%)	0.502
Disease severity score				
APACHEII (points,mean ± SD)	20 ± 7	21 ± 7	20 ± 7	0.422
SOFA (points,median,IQR)	7 (5-11)	7 (5-9)	8 (5-13)	0.162
Treatments during ICU stay				
Tracheal intubation,n (%)	68 (84.0%)	29 (80.6%)	39 (86.7%)	0.457
ECMO,n (%)	15 (18.5%)	7 (19.4%)	8 (17.8%)	0.848
CRRT,n (%)	34 (42.0%)	13 (36.1%)	21 (46.7%)	0.339
Steroid,n (%)	38 (46.9%)	33 (91.7%)	5 (11.1%)	<0.001
Complications and outcomes				
Bacterial infection,n (%)	60 (74.1%)	29 (80.6%)	31 (68.9%)	0.208
Hospital acquired pneumonia,n (%)	49 (60.5%)	21 (58.3%)	28 (62.2%)	0.722
Catheter Related Bloodstream Infection,n (%)	3 (5.2%)	3 (8.3%)	0	0.281
Abdominal infection,n (%)	2 (3.4%)	1 (2.8%)	1 (4.5%)	1.000
Bloodstream infection,n (%)	8 (13.6%)	5 (13.9%)	3 (13.0%)	1.000
Acute renal failure,n (%)	47 (58.0%)	17 (47.2%)	30 (66.7%)	0.078
Circulatory failure,n (%)	36 (44.4%)	19 (52.8%)	17 (37.8%)	0.177
Hepatic failure,n (%)	11 (17.7%)	4 (11.1%)	7 (26.9%)	0.204
Hematological system failure,n (%)	21 (35.6%)	17 (47.2%)	4 (17.4%)	0.040
Central system failure,n (%)	7 (11.5%)	2 (5.6%)	5 (20.0%)	0.183
ICU Mortality,n (%)	51 (63.0%)	24 (66.7%)	27 (60.0%)	0.537
Length of invasive ventilation (only ventilation,days,median,IQR)	12 (5-26) n=66	12 (5-26) n=29	7 (6-31) n=37	0.969
Length of ICU (days,median,IQR)	12 (7-26)	12 (6-26)	13 (7-27)	0.842
Length of hospitalization (days,median,IQR)	20 (10-37)	**26 (13-40)**	18 (10-36)	**0.135**

IQR, Interquartile Range; BMI, Body Mass Index; SD, Standard Deviation; WBC, white blood cell; LYM, lymphocyte; CRP, C-reactive protein; PCT, procalcitonin; ESR, erythrocyte sedimentation rate; APACHE, Acute Physiology and Chronic Health Evaluation; SOFA, sequential organ failure assessment; ECMO, extracorporeal membrane oxygenation; CRRT, continuous renal replacement therapy; Circulatory failure: dopamine>5 or epinephrine>0.1 or norepinephrine>0.1; Hepatic failure: bilirubin>5.9mg/dL (101 μmol/L); Hematological system failure:platelet<50*10^9/L; Central system failure: Glasgow Coma Scale ≤ 9 points.

#### 3.2.3 Multivariate logistic regression analysis for independent risk factors for CAPA in COVID-19 patients admitted to ICU

We included solid organ transplantation, antiviral drugs, antibacterial drugs, total dosage of steroids in the past month for COVID-19, APACHE II scores, SOFA scores, fever, IL-6, IL-8, CD4+ T lymphocytes, CD8+ T lymphocytes, and bronchial wall thickening in the multivariate logistic regression analysis. We found solid organ transplantation [odds ratio (OR) 4.006; 95% confidence interval (CI) 1.014-15.829; P=0.048], APACHEII score ≥20 points [OR 4.331; 95% CI 1.154-16.225; P=0.030], 5 points ≤SOFA score <10 points [OR 4.419; 95% CI 1.215-16.074; P =0.024] were independent risk factors for CAPA (**Table 4**).

**Table 4:** Multivariate logistic Regression Analysis of independent factors associated with CAPA.

	OR	p
Solid organ transplantation	4.006 (1.014-15.829)	0.048
APACHEII score≥20 points	4.331 (1.154-16.255)	0.030
5points≤SOFA score<10points	4.419 (1.215-16.074)	0.024

APACHE, Acute Physiology and Chronic Health Evaluation; SOFA, sequential organ failure assessment.

### 3.3 Significance of microbiological tests for CAPA diagnosis

#### 3.3.1 Microbiological specimens and microbiological examinations of COVID-19 patients

**Figure 3:**
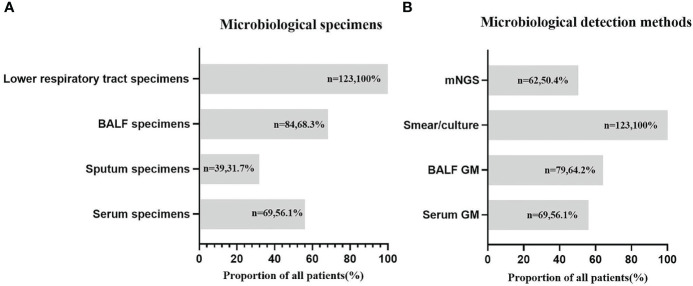
Microbiological specimens **(A)** and microbiological detection methods **(B)** in COVID-19 patients. N—number of COVID-19 patients; %—the proportion of patients from the total number of CAPA patients.

Lower respiratory tract specimens were collected from all patients. BALF specimens were collected from 84/123 (68.3%) patients, and eligible sputum specimens were collected from 39/123 (31.7%) patients. Serum was collected from 69/123 (56.1%) patients. In total, all patients underwent fungal smears and cultures, 79/123 (64.2%) patients had BALF GM detections, 62/123 (50.4%) patients were tested for BALF mNGS; while the 69/123 (56.1%) patients performed serum GM (**Figure 3**). Smear/culture results were positive in 18 patients who were all diagnosed with CAPA. The serum GM test results were positive in 14 patients and 11 of them were ultimately diagnosed with CAPA. Among them, the serum GM index of three patients was between 0.5-0.7 at the initial test. One patient’s serum GM index reduced to normal without antifungal therapy within 5 day and the condition of the other two patients improved without antifungal therapy. These three patients were excluded from the CAPA group. BALF GM was positive in 41 patients, while 28 patients were diagnosed with CAPA, the other 13 patients were excluded from the CAPA group as their BALF GM index reduced to normal without antifungal therapy within 5 days. mNGS were positive in 20 patients, and 19 of them were diagnosed with CAPA, the other one patient was excluded due to negative GM as well as smear/culture results.

#### 3.3.2 Diagnostic performance of four microbiological examinations

**Figure 4:**
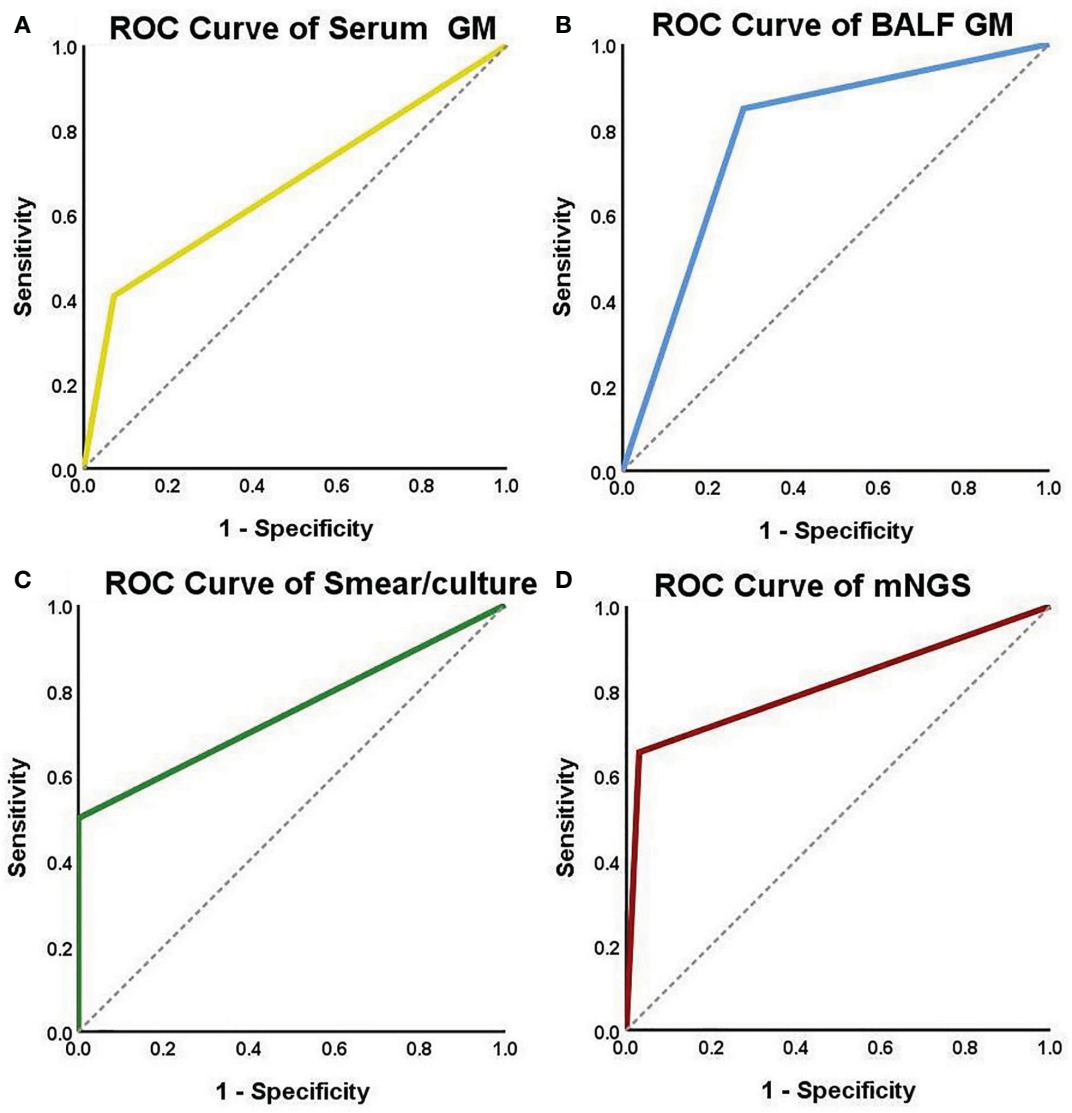
ROC curves for serum GM, BALF GM, smear/culture and mNGS detection. The areas under the ROC curve were 0.668 for the serum GM test **(A)**, 0.783 for the BALF GM test **(B)**, 0.750 for the smear/culture test **(C)**, and 0.812 for the mNGS test **(D)**.

The diagnostic performances of the four microbiological examinations (serum GM >0.5, BALF GM ≥1, positive fungal smear/culture, and positive mNGS results) were explored. The results showed that BALF GM had the highest diagnostic sensitivity (84.9%), followed by mNGS (65.5%), whereas serum GM (40.7%) and fungal smears/cultures (50.0%) had lower sensitivities. The specificity of diagnosis was 100% for fungal culture, followed by mNGS (97.0%), serum GM (79.7%), and BALF GM (71.7%). The positive predictive value was 100% for fungal smear/culture, followed by mNGS (95.0%), serum GM (78.6%), and BALF GM (68.3%). The negative predictive value was highest for BALF GM (86.8%), followed by smear/culture (82.9%), mNGS (76.2%), and serum GM (70.9%). The performances of serum GM, BALF GM, fungal culture, and mNGS were assessed using ROC curve analysis. The area under the curve of the mNGS tests were the highest (0.812), followed by those of the BALF GM tests (0.783), positive fungal smear/culture (0.750), and serum GM tests (0.668) (**Table 5**, **Figure 4**).

**Table 5:** Diagnostic performance of Serum GM, BALF GM, Smear/Culture, mNGS.

	Sensitivity	Specificity	Youden’s index	PPV	NPV
Serum GM	40.7%	79.7%	0.205	78.6%	70.9%
BALF GM	84.9%	71.7%	0.566	68.3%	86.8%
Smear/Culture	50.0%	100.0%	0.500	100.0%	82.9%
mNGS	65.5%	97.0%	0.625	95.0%	76.2%

PPV, positive predictive value; PPV, negative predictive value.

**Figure 5:**
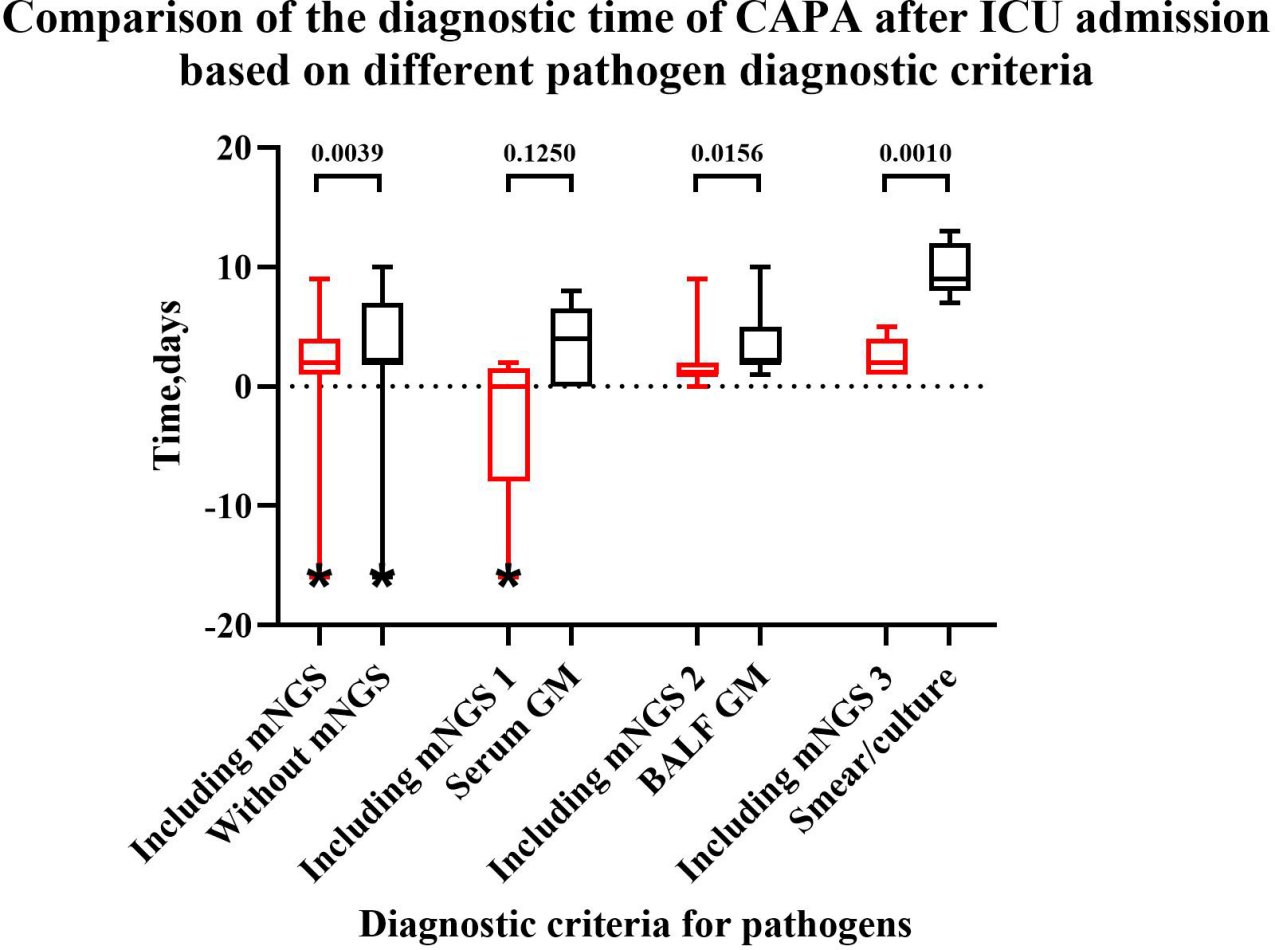
Comparison of the diagnostic time for CAPA after ICU admission based on different pathogen diagnostic criteria. “*” a patient was diagnosed with CAPA before admission to the ICU, resulting in a negative value. “Including mNGS”: Patients with positive mNGS when diagnostic criteria for pathogens included mNGS, serum GM, BALF GM, smear/culture. “Without mNGS”: Patients with positive mNGS when diagnostic criteria for pathogens included serum GM, BALF GM, smear/culture but without mNGS. “Including mNGS 1”: Patients with positive serum GM and mNGS when diagnostic criteria for pathogens included mNGS, serum GM, BALF GM, smear/culture. “Including mNGS 2”: Patients with positive BALF GM and mNGS when diagnostic criteria for pathogens included mNGS, serum GM, BALF GM, smear/culture. “Including mNGS 3”: Patients with positive smear/culture and mNGS when diagnostic criteria for pathogens included mNGS, serum GM, BALF GM, smear/culture. After adding positive mNGS to pathogen diagnostic criteria, the diagnostic time can be shortened compared to without mNGS (p=0.0039), BALF GM (p=0.0156) and smear/culture (p=0.0010).

Among 36 CAPA patients, a total of 19 patients had positive mNGS results. The comparison of diagnostic times of CAPA after ICU admission based on different pathogen diagnostic criteria is presented in **Figure 5**. The results indicate that NGS can facilitate early diagnosis. If the positive result of mNGS is used for CAPA diagnosis, it can lead to a diagnosis up to 1.5 ± 2.3 days earlier (p=0.0036). Among the five CAPA patients with both positive mNGS and serum GM results, the mNGS results were on average 2.8 ± 3.3 days earlier (p=0.1250) than the serum GM results. Among the 15 CAPA patients with positive mNGS and BALF GM results, the mNGS results were on average 1.7 ± 3.1 days earlier (p=0.0156) than the BALF GM results. Furthermore, among the 11 CAPA patients with positive results for fungal culture and mNGS, the diagnosis of CAPA relying on mNGS was 6.7 ± 2.6 days earlier (p=0.0010) than the traditional fungal smear/culture.

## 4 Discussion

CAPA is a common complication in critically ill patients with COVID-19. We found that 29.3% of COVID-19 patients combined with Aspergillus infection in our study. Studies have reported that the mortality rate of CAPA patients can reach 40%-90% (Janssen et al., 2021; Segrelles-Calvo et al., 2021; van Grootveld et al., 2021; Bentvelsen et al., 2022). Most studies have indicated that CAPA patients have a higher mortality rate than non-CAPA patients; while few previous studies have shown no significant difference between the two groups (Calderón-Parra et al., 2022b; Leistner et al., 2022).Although we did not find a significant difference between CAPA and non-CAPA, the mortality rate of CAPA patients in our study is as high as 64.9%. In addition, patients with CAPA required more advanced respiratory support and had more comorbidities and longer ICU stay, which was in accordance with other studies (Calderón-Parra et al., 2022a; Calderón-Parra et al., 2022b; Er et al., 2022; Huang et al., 2022). Therefore, the timely diagnosis and treatments are crucial for the prognosis of critically ill patients with CAPA.

In a multivariate analysis, we found that solid organ transplantation was an independent risk factor for CAPA, which was also found in the univariate results of another study (Calderón-Parra et al., 2022b). Patients who underwent transplantation during the COVID-19 epidemic were immunosuppressed due to heavy postoperative steroid pulse therapy and the use of immunosuppressive drugs, which are risk factors for aspergillosis infection (Huang et al., 2022; Kim et al., 2022; Leistner et al., 2022; Permpalung et al., 2022). We also found APACHEII scores ≥20 points and 5points≤SOFA score<10points points were independent risk factors for CAPA. Previous studies have suggested that patients with CAPA had higher APACHEII scores than non-CAPA patients in univariate analyses (Calderón-Parra et al., 2022a; Calderón-Parra et al., 2022b; Er et al., 2022); the same is true for SOFA scores(Er et al., 2022; Huang et al., 2022). These two scores can be used as indicators to determine the severity of patients with COVID-19 admitted to the ICU. The univariate analysis of our study showed that CAPA patients had higher IL-6 levels, higher IL-8 levels, and lower lymphocyte counts, suggesting that COVID-19 patients might have intensive cytokine storm and the virus may destroy the body’s immune cells, resulting in low immunity and much more susceptibility to fungi (Calderón-Parra et al., 2022a; Ao et al., 2023). In fact, COVID-19 could destroy the patient’s alveolar epithelial-endothelial structure and reduce the antifungal immunity; meanwhile, steroid was the crucial therapy in critically ill COVID-19 patients, all of which increase the risk of infection with other pathogens (Ao et al., 2023), suggesting that COVID-19 infection could serve as a host factor for Aspergillus infection in patients admitted to the ICU (Chiu and Miller, 2019). Furthermore, the combination of Aspergillus infections may increase disease severity in patients with COVID-19.

Routine laboratory tests and chest CT signs did not help with the diagnosis of CAPA. Therefore, the diagnosis of CAPA was highly dependent on microbiological evidence (Chiu and Miller, 2019; Koehler et al., 2021). In terms of respiratory tract specimen collection, considering that the poor sensitivity specificity of a positive Aspergillus culture identified in sputum and ETA and may indicate colonization (Chong and Neu, 2021), it is necessary to undergo bronchoscopy to obtain BALF (Koehler et al., 2021) as early as possible in patients without contraindications, which also helps to identify patients with invasive aspergillus tracheobronchitis simultaneously. Among the microbiological diagnostic methods for CAPA, the positive rate of BALF smear and culture is very low, about 29% to 50%, and the positive rate of sputum specimens is even lower (Zhou et al., 2017; Ullmann et al., 2018). As an antigen released during the growth process of Aspergillus, GM index is an important detection method in the diagnosis of invasive Aspergillus infection. Multiple meta-analyses over the past decade or so have consistently shown that BALF GM has high diagnostic efficacy. It has been reported that BALF GM has good sensitivity[0.90 (95% CI, 0.79-0.96)] and specificity [0.94 (95% CI, 0.90-0.96)] for probable and proven invasive Aspergillosis, which is more powerful than serum GM (Guo et al., 2010). Another study has indicated that the diagnostic efficacy of BALF GM is highest at a cut-off of 0.67 for chronic pulmonary aspergillosis, with a sensitivity of 0.68 (95% CI: 0.51-0.82) and specificity of 0.84 (95% CI: 0.70-0.92), and an AUC of 0.814, which is higher than Serum GM at a cut-off of 0.96, which had an AUC of 0.529 (with a CI: [0.415-0.682][0.307-0.713]), a sensitivity of 0.29 (95% CI: 0.14-0.51), and a specificity of 0.88 (95% CI: 0.73-0.95) (De Oliveira et al., 2023). Previous studies also have shown that the sensitivity of BALF GM (42-100%) is higher than serum GM (3-50%) in patients with relatively normal immune(Chong and Neu, 2021). In addition, the incidence of airway invasion in patients with CAPA was higher than that of vascular invasion, so BALF GM was more meaningful (Zhou et al., 2017). The combined use of blood G and GM was very useful in confirming the diagnosis of IPA and help to identify false positive results (Lu et al., 2011). The results of our study are similar, among all microbiological examinations, BALF GM had the highest sensitivity and serum GM had the lowest sensitivity.

mNGS can also be used to study the physiological mechanism and drug resistance mechanism of some respiratory diseases (such as empyema) (Chen et al., 2021). It has many advantages in fungal detection, such as comprehensive detection of multiple infections, reducing diagnostic time, less affected by prior antibiotic exposure and so on. But it also has many limitations, such as difficulty in distinguishing colonization from infection, and low efficiency of nucleic acid extraction (Deng et al., 2023). mNGS can improve the sensitivity of clinical pathogen detection (Gaston et al., 2022). Ao et al. indicated that in detecting the Aspergillus spp, BALF GM test (57.7%) had the highest sensitivity, followed by mNGS (42.3%), culture (30.8%), serum GM test (26.9%), and smear (7.7%); while mNGS, culture, smear and serum GM test had the specificity of 100%, followed by BALF GM test, with the specificity of 92.9% (Ao et al., 2023) in the community-acquired pneumonia patients. In a study on plasma mNGS in CAPA patients, the sensitivity of mNGS detection for Aspergillus was 67%, and the specificity was 97%(Hoenigl et al., 2023). Our study also found that with the specificity and sensitivity of 62.5% and 97.0%, meanwhile, with a quick report, mNGS might be valuable in timely and accuracy in diagnosis of CAPA. Although there was no significant difference in the diagnostic timing between mNGS and serum GM, this may be due to the small sample size that simultaneously met the criteria for positive blood GM and mNGS.

This study is the largest clinical study to explore the risk factors and the role of microbiological examinations of critically ill CAPA in ICU after the epidemic of Omicron in the mainland China. The biggest advantage of our study was that all patients have obtained qualified lower respiratory tract specimens which were sent for numerous microbiological detections, including fungal smears, cultures, GM detection and mNGS. Meanwhile, BALF specimens were collected from 84/123 (68.3%) patients, and the detection rate of BALF GM and BALF mNGS were 64.2% and 50.4% respectively, improving the sensitivity for the diagnosis. We realize that our study had limitations. First, this is a retrospective single center study with a small number of enrolled patients; second, not all patients undergone the above detections, which might lead to diagnostic bias. Prospective, multi-center studies are desired in order to elucidate the risk factors, clinical presentations and the diagnostic value of microbiological detections of CAPA.

## 5 Conclusions

The incidence of CAPA was extremely high in patients admitted to the ICU. Clinical characteristics, routine laboratory tests, and CT scan features cannot help in identifying CAPA; therefore, the diagnosis of CAPA is highly dependent on microbiological evidence and should be obtained as soon as possible. BALF-GM is the most suitable microbiological examinations for the diagnosis of CAPA. mNGS could assist in early diagnosis and might be an option in critically ill CAPA patients.

## Data availability statement

The raw data supporting the conclusions of this article will be made available by the authors, without undue reservation.

## Ethics statement

Ethical approval was not required for the studies on humans in accordance with the local legislation and institutional requirements because only commercially available established cell lines were used.

## Author contributions

XZ: Conceptualization, Data curation, Investigation, Methodology, Software, Writing – original draft, Formal Analysis, Visualization. XW: Conceptualization, Investigation, Methodology, Software, Writing – original draft, Formal Analysis, Visualization. QZ: Conceptualization, Funding acquisition, Investigation, Methodology, Project administration, Resources, Supervision, Writing – review and editing. LH: Conceptualization, Formal Analysis, Investigation, Methodology, Resources, Supervision, Validation, Visualization, Writing – original draft, Writing – review and editing. ZC: Data curation, Investigation, Methodology, Writing – review and editing. XC: Data curation, Investigation, Methodology, Software, Writing – review and editing. YC: Data curation, Investigation, Methodology, Software, Writing – review and editing. YL: Data curation, Investigation, Methodology, Software, Writing – review and editing. BW: Data curation, Investigation, Methodology, Software, Writing – review and editing.

## References

[B1] Abdolrasouli A. Rhodes J. L. (2022). Phenotypic variants of azole-resistant aspergillus fumigatus that co-exist in human respiratory samples are genetically highly related. Mycopathologia 187 (5-6), 497–508. doi: 10.1007/s11046-022-00665-2 36098829 PMC9469045

[B2] Ao Z. Xu H. Li M. Liu H. Deng M. Liu Y. (2023). Clinical characteristics, diagnosis, outcomes and lung microbiome analysis of invasive pulmonary aspergillosis in the community-acquired pneumonia patients. BMJ Open Respir. Res. 10 (1), e001358. doi: 10.1136/bmjresp-2022-001358 PMC9972439 36828645

[B3] Argenziano M. G. Bruce S. L. Slater C. L. Tiao J. R. Baldwin M. R. Barr R. G.. (2020). Characterization and clinical course of 1000 patients with coronavirus disease 2019 in New York: retrospective case series. BMJ 369, m1996. doi: 10.1136/bmj.m1996 32471884 PMC7256651

[B4] Auld S. C. Caridi-Scheible M. Blum J. M. Robichaux C. Kraft C. Jacob J. T.. (2020). ICU and ventilator mortality among critically ill adults with coronavirus disease 2019. Crit. Care Med. 48 (9), e799–e804. doi: 10.1097/CCM.0000000000004457 32452888 PMC7255393

[B5] Autier B. Prattes J. White P. L. Valerio M. MaChado M. Price J.. (2022). Aspergillus lateral flow assay with digital reader for the diagnosis of COVID-19-associated pulmonary aspergillosis (CAPA): a multicenter study. J. Clin. Microbiol. 60 (1), e0168921. doi: 10.1128/JCM.01689-21 34643415 PMC8769727

[B6] Bao S. Song H. Chen Y. Zhong C. Tang H. (2022). Metagenomic next-generation sequencing for the diagnosis of pulmonary aspergillosis in non-neutropenic patients: a retrospective study. Front. Cell. infection Microbiol. 12. doi: 10.3389/fcimb.2022.925982 PMC9376315 35979088

[B7] Bellanger A. P. Lallemand S. Tumasyan H. A. Navellou J. C. Barrera C. Rouzet A.. (2022). Investigation of the value of precipitins in severe acute respiratory syndrome coronavirus 2 (SARS-CoV-2) patients with a positive marker for Aspergillus species. Med. Mycol 60 (5), myac031. doi: 10.1093/mmy/myac031 35604675 PMC9213863

[B8] Bentvelsen R. G. Arkel A. Rijpstra T. A. Kant M. K. M. Brugge S. V. S. Loth D. W.. (2022). Regional impact of COVID-19-associated pulmonary aspergillosis (CAPA) during the first wave. J. Fungi (Basel) 8 (2), 96. doi: 10.3390/jof8020096 35205851 PMC8875881

[B9] Boyd S. Martin-Loeches I. (2021). Rates of aspergillus co-infection in COVID patients in ICU not as high as previously reported. Clin. Infect. Dis. 73 (5), e1236–e1238. doi: 10.1093/cid/ciab008 33417690

[B10] Calderón-Parra J. Mills-Sanchez P. Moreno-Torres V. Tejado-Bravo S. Romero-Sánchez I. Balandin-Moreno B.. (2022a). COVID-19-associated pulmonary aspergillosis (CAPA): Risk factors and development of a predictive score for critically ill COVID-19 patients. Mycoses 65 (5), 541–550. doi: 10.1111/myc.13434 35212030 PMC9115267

[B11] Calderón-Parra J. Moreno-Torres V. Mills-Sanchez P. Tejado-Bravo S. Romero-Sánchez I. Balandin-Moreno B.. (2022b). Association of COVID-19-associated pulmonary aspergillosis with cytomegalovirus replication: A case-control study. J. Fungi (Basel) 8 (2), 161. doi: 10.3390/jof8020161 35205914 PMC8877274

[B12] Cao J. Tu W. J. Cheng W. Yu L. Liu Y. K. Hu X.. (2020). Clinical features and short-term outcomes of 102 patients with coronavirus disease 2019 in wuhan, China. Clin. Infect. Dis. 71 (15), 748–755. doi: 10.1093/cid/ciaa243 32239127 PMC7184479

[B13] Chen Z. Cheng H. Cai Z. Wei Q. Li J. Liang J.. (2021). Identification of microbiome etiology associated with drug resistance in pleural empyema. Front. Cell Infect. Microbiol. 11. doi: 10.3389/fcimb.2021.637018 PMC8008065 33796482

[B14] Chen L. Han X. Li Y. Zhang C. Xing X. (2020a). Invasive pulmonary aspergillosis in immunocompetent patients hospitalised with influenza A-related pneumonia: a multicenter retrospective study. BMC Pulm Med. 20 (1), 239. doi: 10.1186/s12890-020-01257-w 32907585 PMC7479745

[B15] Chen L. Xu Y. Liu C. Huang H. Zhong X. Ma C.. (2020b). Clinical features of aseptic meningitis with varicella zoster virus infection diagnosed by next-generation sequencing: case reports. BMC Infect. Dis. 20 (1), 1–435. doi: 10.1186/s12879-020-05155-8 PMC7309994 32571239

[B16] Chen N. Zhou M. Dong X. Qu J. Gong F. Han Y.. (2020c). Epidemiological and clinical characteristics of 99 cases of 2019 novel coronavirus pneumonia in Wuhan, China: a descriptive study. Lancet 395 (10223), 507–513. doi: 10.1016/S0140-6736(20)30211-7 32007143 PMC7135076

[B17] Chiu C. Y. Miller S. A. (2019). Clinical metagenomics. Nat. Rev. Genet. 20 (6), 341–355. doi: 10.1038/s41576-019-0113-7 30918369 PMC6858796

[B18] Chong W. H. Neu K. P. (2021). Incidence, diagnosis and outcomes of COVID-19-associated pulmonary aspergillosis (CAPA): a systematic review. J. Hosp. infection 113, 115–129. doi: 10.1016/j.jhin.2021.04.012 PMC8057923 33891985

[B19] Coste A. Frérou A. Raute A. Couturaud F. Morin J. Egreteau P. Y.. (2021). The extent of aspergillosis in critically ill patients with severe influenza pneumonia: A multicenter cohort study. Crit. Care Med. 49 (6), 934–942. doi: 10.1097/CCM.0000000000004861 33591000

[B20] Deng W. Jiang Y. Qin J. Chen G. Lv Y. Lei Y.. (2023). Metagenomic next-generation sequencing assists in the diagnosis of mediastinal aspergillus fumigatus abscess in an immunocompetent patient: A case report and literature review. Infect. Drug Resist. 16, 1865–1874. doi: 10.2147/IDR.S399484 37020798 PMC10069495

[B21] De Oliveira V. F. Silva G. D. Taborda M. Levin A. S. Magri M. M. C. (2023). Systematic review and meta-analysis of galactomannan antigen testing in serum and bronchoalveolar lavage for the diagnosis of chronic pulmonary aspergillosis: defining a cutoff. Eur. J. Clin. Microbiol. Infect. Dis. 42 (9), 1047–1054. doi: 10.1007/s10096-023-04639-0 37430166

[B22] Docherty A. B. Harrison E. M. Green C. A. Hardwick H. E. Pius R. Norman L.. (2020). Features of 20 133 UK patients in hospital with covid-19 using the ISARIC WHO Clinical Characterisation Protocol: prospective observational cohort study. BMJ 369, m1985–m1985. doi: 10.1136/bmj.m1985 32444460 PMC7243036

[B23] Du R. H. Liu L. M. Yin W. Wang W. Guan L. L. Yuan M. L.. (2020). Hospitalization and critical care of 109 decedents with COVID-19 pneumonia in wuhan, China. Ann. Am. Thorac. Soc. 17 (7), 839–846. doi: 10.1513/AnnalsATS.202003-225OC 32255382 PMC7328178

[B24] Er B. Er A. G. Gülmez D. Şahin T. K. Halaçlı B. Durhan G.. (2022). A screening study for COVID-19-associated pulmonary aspergillosis in critically ill patients during the third wave of the pandemic. Mycoses 65 (7), 724–732. doi: 10.1111/myc.13466 35531631 PMC9348343

[B25] Erami M. Hashemi S. J. Raiesi O. Fattahi M. Getso M. I. Momen-Heravi M.. (2023). COVID-19-associated pulmonary aspergillosis (CAPA) in Iranian patients admitted with severe COVID-19 pneumonia. Infection 51 (1), 223–230. doi: 10.1007/s15010-022-01907-7 36107379 PMC9476444

[B26] Ergün M. Brüggemann R. J. M. Alanio A. Dellière S. van Arkel A. Bentvelsen R. G.. (2021). Aspergillus test profiles and mortality in critically ill COVID-19 patients. J. Clin. Microbiol. 59 (12), e0122921. doi: 10.1128/JCM.01229-21 34495710 PMC8601217

[B27] Gaston D. C. Miller H. B. Fissel J. A. Jacobs E. Gough E. Wu J.. (2022). Evaluation of metagenomic and targeted next-generation sequencing workflows for detection of respiratory pathogens from bronchoalveolar lavage fluid specimens. J. Clin. Microbiol. 60 (7), e0052622. doi: 10.1128/jcm.00526-22 35695488 PMC9297812

[B28] Guo Y.-L. Chen Y.-Q. Wang K. Qin S.-M. Wu C. Kong J.-L. (2010). Accuracy of BAL galactomannan in diagnosing invasive aspergillosis. Chest 138 (4), 817–824. doi: 10.1378/chest.10-0488 20453070

[B29] Hoenigl M. Egger M. Price J. Krause R. Prattes J. White P. L. (2023). Metagenomic next-generation sequencing of plasma for diagnosis of COVID-19-associated pulmonary aspergillosis. J. Clin. Microbiol. 61 (3), e0185922–e0185922. doi: 10.1128/jcm.01859-22 36809121 PMC10035327

[B30] Huang J. R. Shen H. C. Sun C. Y. Chen W. C. Chen Y. M. Feng J. Y.. (2022). COVID-19-associated pulmonary aspergillosis is associated with increased in-hospital mortality and prolonged SARS-CoV-2 viral shedding. J. Formos Med. Assoc. 121 (12), 2617–2625. doi: 10.1016/j.jfma.2022.07.006 35953342 PMC9359693

[B31] Janssen N. A. F. Nyga R. Vanderbeke L. Jacobs C. Ergün M. Buil J. B.. (2021). Multinational observational cohort study of COVID-19-associated pulmonary aspergillosis(1). Emerg. Infect. Dis. 27 (11), 2892–2898. doi: 10.3201/eid2711.211174 34519638 PMC8544971

[B32] Jiang Z. Chen S. Zhu Q. Xiao Y. Qu J. (2022). COVID-19-associated pulmonary aspergillosis in a tertiary care center in Shenzhen City. J. infection Public Health 15 (2), 222–227. doi: 10.1016/j.jiph.2021.12.015 35032951 PMC8733224

[B33] Kim S. H. Hong J. Y. Bae S. Lee H. Wi Y. M. Ko J. H.. (2022). Risk factors for coronavirus disease 2019 (COVID-19)-associated pulmonary aspergillosis in critically ill patients: A nationwide, multicenter, retrospective cohort study. J. Korean Med. Sci. 37 (18), e134. doi: 10.3346/jkms.2022.37.e134 35535369 PMC9091428

[B34] Koehler P. Bassetti M. Chakrabarti A. Chen S. C. A. Colombo A. L. Hoenigl M.. (2021). Defining and managing COVID-19-associated pulmonary aspergillosis: the 2020 ECMM/ISHAM consensus criteria for research and clinical guidance. Lancet Infect. Dis. 21 (6), e149–e162. doi: 10.1016/S1473-3099(20)30847-1 33333012 PMC7833078

[B35] Leistner R. Schroeter L. Adam T. Poddubnyy D. Stegemann M. Siegmund B.. (2022). Corticosteroids as risk factor for COVID-19-associated pulmonary aspergillosis in intensive care patients. Crit. Care 26 (1), 30. doi: 10.1186/s13054-022-03902-8 35090528 PMC8796178

[B36] Li Y. Sun B. Tang X. Liu Y. L. He H. Y. Li X. Y.. (2020). Application of metagenomic next-generation sequencing for bronchoalveolar lavage diagnostics in critically ill patients. Eur. J. Clin. Microbiol. Infect. Dis. 39 (2), 369–374. doi: 10.1007/s10096-019-03734-5 31813078 PMC7102353

[B37] Lu Y. Chen Y.-Q. Guo Y.-L. Qin S.-M. Wu C. Wang K. (2011). Diagnosis of invasive fungal disease using serum (1right arrow3)-[beta]-D-glucan: A bivariate meta-analysis. Internal Med. 50 (22), 2783–2791. doi: 10.2169/internalmedicine.50.6175 22082890

[B38] Machado M. Valerio M. Álvarez-Uría A. Olmedo M. Veintimilla C. Padilla B.. (2021). Invasive pulmonary aspergillosis in the COVID-19 era: An expected new entity. Mycoses 64 (2), 132–143. doi: 10.1111/myc.13213 33210776 PMC7753705

[B39] Permpalung N. Chiang T. P. Massie A. B. Zhang S. X. Avery R. K. Nematollahi S.. (2022). Coronavirus disease 2019-associated pulmonary aspergillosis in mechanically ventilated patients. Clin. Infect. Dis. 74 (1), 83–91. doi: 10.1093/cid/ciab223 33693551 PMC7989534

[B40] Prattes J. Wauters J. Giacobbe D. R. Salmanton-García J. Maertens J. Bourgeois M.. (2022). Risk factors and outcome of pulmonary aspergillosis in critically ill coronavirus disease 2019 patients-a multinational observational study by the European Confederation of Medical Mycology. Clin. Microbiol. Infect. 28 (4), 580–587. doi: 10.1016/j.cmi.2021.08.014 34454093 PMC8387556

[B41] Schauwvlieghe A. Rijnders B. J. A. Philips N. Verwijs R. Vanderbeke L. Van Tienen C.. (2018). Invasive aspergillosis in patients admitted to the intensive care unit with severe influenza: a retrospective cohort study. Lancet Respir. Med. 6 (10), 782–792. doi: 10.1016/S2213-2600(18)30274-1 30076119

[B42] Segrelles-Calvo G. Araújo G. R. S. Llopis-Pastor E. Carrillo J. Hernández-Hernández M. Rey L.. (2021). Prevalence of opportunistic invasive aspergillosis in COVID-19 patients with severe pneumonia. Mycoses 64 (2), 144–151. doi: 10.1111/myc.13219 33217071 PMC7753478

[B43] Song G. Liang G. Liu W. (2020). Fungal co-infections associated with global COVID-19 pandemic: A clinical and diagnostic perspective from China. Mycopathologia 185 (4), 599–606. doi: 10.1007/s11046-020-00462-9 32737747 PMC7394275

[B44] Ullmann A. J. Aguado J. M. Arikan-Akdagli S. Denning D. W. Groll A. H. Lagrou K.. (2018). Diagnosis and management of Aspergillus diseases: executive summary of the 2017 ESCMID-ECMM-ERS guideline. Clin. Microbiol. infection 24, e1–e38. doi: 10.1016/j.cmi.2018.01.002 29544767

[B45] van Grootveld R. van Paassen J. de Boer M. G. J. Claas E. C. J. Kuijper E. J. van der Beek M. T. (2021). Systematic screening for COVID-19 associated invasive aspergillosis in ICU patients by culture and PCR on tracheal aspirate. Mycoses 64 (6), 641–650. doi: 10.1111/myc.13259 33606324 PMC8014245

[B46] Verweij P. E. Rijnders B. J. A. Brüggemann R. J. M. Azoulay E. Bassetti M. Blot S.. (2020). Review of influenza-associated pulmonary aspergillosis in ICU patients and proposal for a case definition: an expert opinion. Intensive Care Med. 46 (8), 1524–1535. doi: 10.1007/s00134-020-06091-6 32572532 PMC7306567

[B47] Wang J. Yang Q. Zhang P. Sheng J. Zhou J. Qu T. (2020). Clinical characteristics of invasive pulmonary aspergillosis in patients with COVID-19 in Zhejiang, China: a retrospective case series. Crit. Care (London England) 24 (1), 299–299. doi: 10.1186/s13054-020-03046-7 PMC7274513 32503617

[B48] WHO (2022) Therapeutics and COVID-19: living guideline. Available at: https://www.who.int/publications/i/item/WHO-2019-nCoV-therapeutics-2022.5 (Accessed September 16th 2022).

[B49] Xu J. Yang X. Lv Z. Zhou T. Liu H. Zou X.. (2021). Risk factors for invasive aspergillosis in patients admitted to the intensive care unit with coronavirus disease 2019: A multicenter retrospective study. Front. Med. 8. doi: 10.3389/fmed.2021.753659 PMC8635191 34869450

[B50] Zhou W. Li H. Zhang Y. Huang M. He Q. Li P.. (2017). Diagnostic value of galactomannan antigen test in serum and bronchoalveolar lavage fluid samples from patients with nonneutropenic invasive pulmonary aspergillosis. J. Clin. Microbiol. 55, 2153–2161. doi: 10.1128/JCM.00345-17 28446576 PMC5483917

